# The Lords' Committee

**Published:** 1891-02-14

**Authors:** 


### The Lords' Committee.

Mr. J. G. Wainwright, Treasurer, was examined on February 5th with regard to the management of St. Thomas's Hospital. The sisters and nurses and other servants were appointed by the Treasurer, and he had also the power of dismissing them; and in fact he exercised paramount authority in the hospital in the absence of the Committee and Almoners. With regard to St. Thomas's Home, the minimum payment was 9s. a head, and in addition they had also paying patients in the wards under the Charity Commissioners' scheme. He did not know whether there was any limit upon them in regard to the number of paying patients they received, but they practically did not get many; and the whole receipts from that source last year was £150. There was a lamentable want of hospital accommodation in the south of London, and he was certain that they could fill many more beds if they had them at St. Thomas's. The out-patients' department was a very large one. In the case of a death in the hospital, the friends of the patient had to see to the funeral arrangements. Earl Cathcart: We were told a very sad thing the other day with regard to your hospital—that a great many poor people were sent away, and it was suggested that that arose from a great part of your income being dissipated by expensive buildings.—The witness said he did not think so. Several other questions were put which implied that the management of St. Thomas's was expensive, and the buildings too far apart. Miss Entwhistle, a former nurse was then called. She was of opinion that the sisters had too much untrained labour under them, and had to do a great deal of work because the probationers did not know how to do it. The same applied to the staff nurses, and she thought there ought to be two day staff nurses instead of one in each ward that contained over thirty beds. Sometimes as many as thirty-five to forty beds were placed in a ward, but no extra nurses were appointed to attend to those extra patients. The nurses and sisters wanted more rest and more holiday. Miss Gordon, the Matron, gave contradictory evidence. There was a staff of 115 nurses. She thought that their work was not too hard, although they were sometimes pushed for nurses, and had to go outside for assistance. They now had under consideration the question of the holidays, the hours of work, and the time of meals. She had talked the matter over with the Treasurer, and they hoped to have a new scheme before the summer holidays. In answer to Lord Monkswell the witness said that, taking into account the time the nurses had off on Sundays, the average number of hours for the week would not be more than sixty. They certainly were not so hard worked as the nurses at the Leeds Hospital, where she had had previous experience. Dr. Sharkey next gave evidence as to the treatment of the patients in both the in and out-patient departments, and expressed the opinion that the out-patient department was certainly not abused, and that the system limiting the number of out-patients worked very well.

On Monday last some eight members of the Committee were present and three or four reporters; Lord Sandhurst sticks nobly to his post as Chairman. Mr. Charles Tod, for twenty years Secretary of St. George's Hospital gave evidence. The wards were often crowded; but there had been no complaints received, although some of the doctors would like the cubic feet to each patient increased by taking out a few beds. That matter was under consideration. Many of the patients admitted were destitute, and they and their wives and families were relieved from the Samaritan Fund. About 15 per cent. of the patients were servants. He thought the hospital accommodation of the neighbourhood was deficient. The same system of nursing was employed as at most of the other hospitals, though they did not call the chief nurses "sisters," but "head nurses." There were ninety-two nurses on the staff, and no cases of diphtheria had occurred among them. They restricted the out-patients to fifteen new cases each day to each medical officer, and very often they were obliged to say that the number of tickets had been given out, and that no more patients could be seen. To work the hospital thoroughly well they wanted more nurses, and there was no room to accommodate them inside the hospital. One of the Lords complained of the dismal outside appearance of St. George's, and asked if it could not be cleaned. Dr. Whipham, Dean of the Medical School at St. George's, stated that the number of students was about 140, and those who entered for the higher curriculum paid £120. The fees made up the income of the school to about £4,500 a year, and out of those fees the lecturers and expenses were paid—the latter being about £2,000. The out-patient department at St. George's had always been very crowded. Those who attended were not able to pay fees to a medical man, and if not seen at the out-patient department of the hospital would have to go to the poor-law. He considered it would be a hardship to the public if the out-patient department were abolished. Dr. Isambard Owen, senior assistant physician of St. George's, stated that, with regard to the fees paid to the lecturers and teachers, none would get more than £100 a year, but the minimum would be about £30. Inquiries were made as to the circumstances of the patients, and if any doubts arose, although if urgent they would receive first treatment, the matter was referred to the Charity Organisation Society. Mr. H. H. Clutton of St. Thomas's stated that the pressure on the surgical side of the out-patient department of the hospital was not excessive. He did not desire the department done away with; he considered the poor outside practitioner was more frightened than hurt.

